# Construction of a Pan-Genome Allele Database of *Salmonella enterica* Serovar Enteritidis for Molecular Subtyping and Disease Cluster Identification

**DOI:** 10.3389/fmicb.2016.02010

**Published:** 2016-12-15

**Authors:** Yen-Yi Liu, Chih-Chieh Chen, Chien-Shun Chiou

**Affiliations:** ^1^Central Regional Laboratory, Center for Diagnostics and Vaccine Development, Centers for Disease ControlTaichung, Taiwan; ^2^Institute of Medical Science and Technology, National Sun Yat-sen UniversityKaohsiung, Taiwan; ^3^Medical Science and Technology Center, National Sun Yat-sen UniversityKaohsiung, Taiwan

**Keywords:** next generation sequencing (NGS), pan-genome allele database, whole genome multilocus sequence typing (wgMLST), typing, molecular epidemiology

## Abstract

We built a pan-genome allele database with 395 genomes of *Salmonella enterica* serovar Enteritidis and developed computer tools for analysis of whole genome sequencing (WGS) data of bacterial isolates for disease cluster identification. A web server (http://wgmlst.imst.nsysu.edu.tw) was set up with the database and the tools, allowing users to upload WGS data to generate whole genome multilocus sequence typing (wgMLST) profiles and to perform cluster analysis of wgMLST profiles. The usefulness of the database in disease cluster identification was demonstrated by analyzing a panel of genomes from 55 epidemiologically well-defined *S.* Enteritidis isolates provided by the Minnesota Department of Health. The wgMLST-based cluster analysis revealed distinct clades that were concordant with the epidemiologically defined outbreaks. Thus, using a common pan-genome allele database, wgMLST can be a promising WGS-based subtyping approach for disease surveillance and outbreak investigation across laboratories.

## Introduction

Characterization of bacterial isolates using various subtyping methods has been a fundamental work for epidemiologic study of infectious diseases. Pulsed-field gel electrophoresis (PFGE) is currently the gold standard of molecular subtyping tool in discriminating between bacterial strains for disease surveillance and outbreak investigation. PFGE has been the common subtyping tool employed in the laboratories of PulseNet, a molecular subtyping network for foodborne bacterial disease surveillance (Swaminathan et al., 2001). However, PFGE is labor-intensive and time-consuming and lacks sufficient resolution for highly clonal bacterial strains (Boxrud et al., 2007; Liang et al., 2007). Over the past two decades, public health laboratories have become eager for a new subtyping tool to provide better resolution than PFGE for examining highly clonal bacterial strains. With the advance of next generation sequencing (NGS) techniques, whole genome sequencing (WGS) has become a rapid and inexpensive method for the characterization of bacteria. WGS-based analysis has been increasingly used in public health laboratories to characterize bacterial pathogens and perform subtyping for epidemiologic study (Deng et al., 2016; Fratamico et al., 2016; Lindsey et al., 2016).

The current second generation sequencing platforms usually generate millions of short sequences (reads) from a bacterial genome. It has been a great challenge to analyze the vast number of short sequences to obtain specific information. Several sequence analysis tools and bioinformatic pipelines have been developed, and some have been installed on the website of the Center for Genome Epidemiology^1^ so WGS data may be used for identification of bacterial species, resistance genes, virulence factors, serotypes, and analysis of phylogeny. However, public health laboratories are in need of tools that can analyze WGS data to generate a type of portable genetic fingerprint (genotype) for use in discriminating among closely related strains for inter-laboratory perspective disease surveillance and outbreak investigation.

The single-nucleotide polymorphism (SNP)-based method is a widely used approach in which WGS data are employed for high-resolution subtyping of bacterial strains (Taylor et al., 2015; Bekal et al., 2016). To apply the SNP-based approach, a reference genome sequence is required for calling SNPs from WGS data of strains. A potential drawback is that the use of different reference genomes between studies would result in different SNP profiles, making it difficult to compare results between studies. Whole genome multilocus sequence typing (wgMLST), an extension of traditional MLST (Maiden et al., 1998), is a genome wide gene-by-gene comparison approach (Maiden et al., 2013). This wgMLST-based approach has been applied to analyze WGS data for detection of disease clusters and outbreak investigation (de Been et al., 2015; Jackson et al., 2016; Jonathan et al., 2016). To make the wgMLST scheme a standard subtyping tool, a pan-genome allele database that contains genes present in the population of a bacterial organism has to be established first. wgMLST profiles generated from a common pan-genome allele database can be portable and comparable across laboratories.

In this study, we constructed an *S.* Enteritidis pan-genome allele database for analysis of WGS data of bacterial isolates. A web server with the database was built, and computer tools were installed in the website to allow users to generate wgMLST profiles and compare genetic relatedness among bacterial isolates. The usefulness of the database in identification of disease clusters was assessed using genomes from a panel of epidemiologically well-characterized *S*. Enteritidis isolates provided by the Minnesota Department of Health.

## Materials and Methods

### Building an *S.* Enteritidis Pan-Genome Allele Database

*Salmonella enterica* serovar Enteritidis pan-genome allele database was constructed with 340 *S.* Enteritidis genomic sequences retrieved from the NCBI Assembly database^2^ and 55 *S.* Enteritidis genomic sequences provided by Minnesota Department of Health^3^ (**Supplementary Table S1**). The 395 complete genomic sequences or genomic contigs were first annotated using Prokka (Seemann, 2014), a rapid bacterial genome annotation pipeline, to generate output gff files. The gff files were processed using the PGAdb_builder, a pipeline for construction of a bacterial pan-genome allele database (Liu et al., 2016). In this study, paralogous genes were excluded from the dataset, and proteins sharing ≥95% amino acid sequence identity were grouped in an orthologous cluster (a protein family). A protein family was assigned to be a locus, and each protein in a locus was transferred back to its nucleotide sequence by referring to the ffn file that was created in the annotation step. Nucleotide sequences in a locus differing by a nucleotide or more from each other are defined as different alleles. Loci and alleles of a pan-genome allele dataset are encoded with a standardized numbering system.

### wgMLST Profiling

The wgMLST_profiling tool was developed to generate wgMLST profiles from bacterial genomes in use of the *S.* Enteritidis pan-genome allele database. A wgMLST_profiling was installed on the website, http://wgmlst.imst.nsysu.edu.tw, to allow users to upload assembled whole genome contigs to generate wgMLST profiles. For profiling, the longest allele (or the first one as two or more allelic sequences having the same length) for each locus was selected to make up a reference sequence set (RSS). Query genomic contigs were compared with the RSS using BLASTN algorithm (Altschul et al., 1990). In the present study, the presence of a locus in a query genome was defined as existence of a sequence that shared ≥90% length coverage and ≥90% sequence identity with one reference sequence of the RSS. Sequences from the query genome were subsequently compared with all allelic sequences in the locus and given a digital number for the locus by a designated numbering system.

### Analysis of Genetic Relatedness

A DendroMaker tool was developed and installed in the web service site for genetic relatedness analysis of bacterial strains. The program used Manhattan distance coefficient and unweighted pair group method with arithmetic mean (UPGMA) algorithm for cluster analysis of wgMLST profiles.

### Web Service

A web server (SE-PGAdb) was built with *S.* Enteritidis pan-genome allele database and wgMLST_Profiling and DendroMaker tools written in PHP scripts. The web page^4^ was constructed in HTML, JavaScript, and PHP formats. The server runs on a Linux cluster with 2.40 GHz Intel Xeon processors comprising 24 cores.

### Input Format

The wgMLST_Profiling module accepts genome contigs in FASTA format. Sequence comparison is performed using the BLASTN algorithm. The maximal number of genomes uploaded for profiling is set at 99. The DendroMaker module accepts wgMLST profiles generated from the wgMLST_Profiling module. The maximal number for constructing a dendrogram is set at 999 wgMLST profiles.

## Results

### *S*. Enteritidis Pan-Genome Allele Database

**FIGURE 1:**
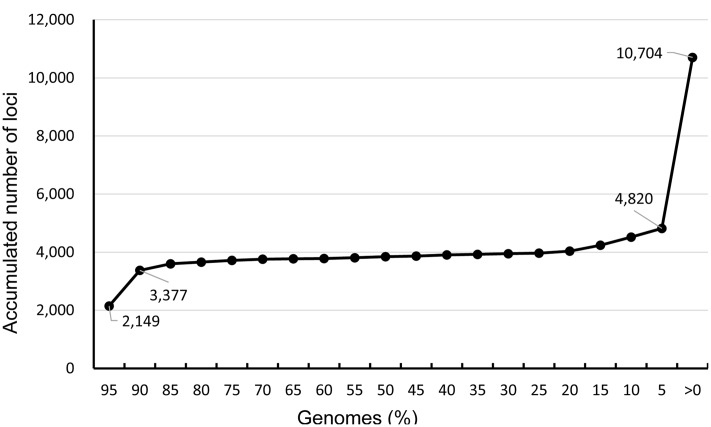
**Distribution of accumulated number of loci over genomes in an *S.* Enteritidis pan-genome database.** The database is constructed with 395 *S.* Enteritidis genomes and contains 10,704 loci (genes) of which 3,377 loci are shared by ≥90% genomes used to make wgMLST profiles.

The database constructed with 395 genomes contained 10,704 loci (genes) of which 2,149 loci were shared by ≥95% of the genomes, 3,377 loci by ≥90% of the genomes, and 4,820 loci by ≥5% of the genomes (**Figure 1**). No genes were shared by all of the genomes, but as many as 2,830 loci (26.4%) existed in only 1 genome. Two loci were most common and shared by 391 (99.0%) genomes. The distribution of number of loci over number of genomes increased and reached a peak at which 225 loci were present in 379 (96.0%) genomes. The number of loci accumulated slowly from 90 to 5% of the genomes, but a large number of loci were present in very few genomes. There were 5,884 (55.0%) loci present in only 1 to 19 (≤ 5%) genomes.

In the present study, core genes were designated to those present in ≥90% of the 395 genomes. Dispensable genes were those present in two or more genomes, but less than 90% of the genomes. Unique genes were those present in one genome. In this database, 31.5% (3,377 loci) belonged to core genes, 42.0% (4,497 loci) dispensable genes, and 26.4% (2,830 loci) unique genes.

### Performance of wgMLST on Identifying Disease Clusters

**FIGURE 2:**
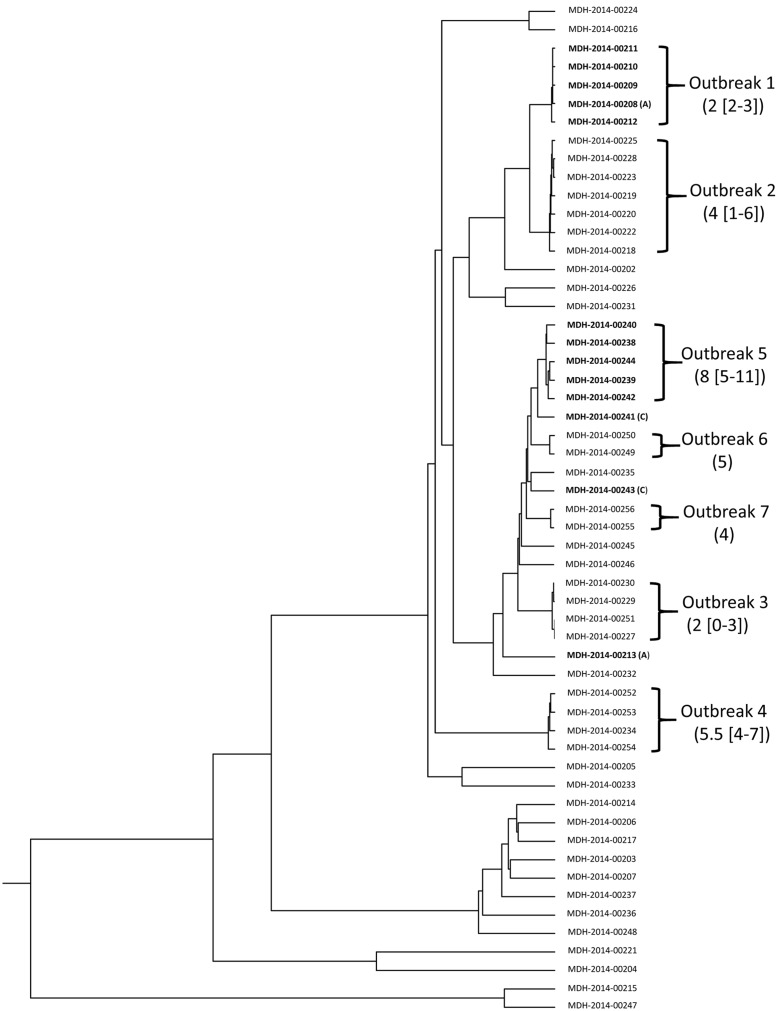
**Dendrogram constructed with wgMLST profiles for 55 epidemiologically well-characterized *S.* Enteritidis isolates from Minnesota Department of Health (Taylor et al., 2015).** The wgMLST profiles were generated based on the 3,377 core genes. The seven outbreaks are marked, and the distance in number of loci (median [range]) among the isolates is labeled. The outbreak 1-suspected isolates are indicated with (A) and the outbreak 5-suspected isolates are indicated with (C).

The *S.* Enteritidis pan-genome allele database was applied to generate wgMLST profiles for 55 epidemiologically well-defined *S.* Enteritidis isolates from the Minnesota Department of Health. The panel included isolates from seven outbreaks and epidemiologically unrelated isolates, and the genetic relatedness among the isolates had been analyzed using the SNP-based approach (Taylor et al., 2015). Cluster analysis of wgMLST profiles generated a genetic relatedness tree which had a dendrogram topology highly similar to that constructed with the SNP profiles (Taylor et al., 2015). The dendrogram revealed distinct clades that were concordant with the seven disease outbreaks (**Figure 2**). Isolates for each outbreak varied by 0 to 11 loci. Consistent with the SNP-based analysis, wgMLST-based analysis excluded one of the 2 outbreak 1-suspected isolates (MDH-2014-00213) and 2 outbreak 5-suspected isolates (MDH-2014-00241 and MDH-2014-00243) from the related outbreaks (**Figure 2**). MDH-2014-00213 had a distance of 92–95 loci to the outbreak 1 isolates. The outbreak 5-suspected isolates were genetically close to outbreak 5 isolates; MDH-2014-00241 and MDH-2014-00243 had a distance of 17–19 loci and 23–30 loci to the outbreak 5 isolates, respectively.

## Discussion

We constructed an *S.* Enteritidis pan-genome allele database and developed a wgMLST_profiling tool to analyze WGS data for disease cluster identification. Using a pan-genome allele database, the wgMLST_profiling program generates wgMLST profiles that can be used to assess the genetic relatedness between bacterial strains. The evaluation with 55 epidemiologically well-defined isolates from the Minnesota Department of Health indicates that wgMLST has a high resolution in discriminating between isolates for identification of disease clusters. Another advantage is that the wgMLST file is relatively small in size and portable; it can be a promising WGS-based standard subtyping tool for disease surveillance and outbreak investigation across laboratories.

The wgMLST approach has been used as a subtyping tool in the epidemiological study of various bacterial pathogens (Cody et al., 2013; Bratcher et al., 2014; Kohl et al., 2014; Leopold et al., 2014; Antwerpen et al., 2015; de Been et al., 2015; Jackson et al., 2016; Lindsey et al., 2016; Raphael et al., 2016). This gene-by-gene comparison method is able to provide high-resolution typing results to allow accurate discrimination between epidemiologically related isolates and unrelated isolates. wgMLST uses the same principle as the SNP-based approach; it converts SNPs in target genes into a standardized and portable allele numbering system. Our study and others indicate that the performance of wgMLST is equivalent to that of the SNP-based approach (de Been et al., 2015; Raphael et al., 2016). One great advantage is that the wgMLST profile consists of serial digital numbers in numeric order that represent alleles of the target genes. Thus, wgMLST is far less computationally intensive than an SNP-based approach for using WGS data to investigate genetic relatedness among bacterial strains.

The *S.* Enteritidis pan-genome allele database constructed with 395 genomes comprises 10,704 loci of which 31.5% (3,377 loci) exist in ≥90% of the genomes, only 13.5% (1,443 loci) are distributed in 5 to 90% of the genomes, and 55% (5,884 loci) are present in 1 to 19 (5%) of the genomes. Theoretically, the more target genes used for comparison, the higher the resolution in discriminating between bacterial strains. In the present study, we used the 3,377 loci (core genes) to be the target genes for wgMLST profiling. Our evaluation with the panel of epidemiologically well-defined *S.* Enteritidis isolates indicates that wgMLST profiling based on the 3,377 loci is able to provide sufficient resolution in discerning the outbreak isolates from non-outbreak isolates. We also compared the performance of wgMLST by profiling from 2,149 loci (distributed in ≥95% of the genomes), 3,602 loci (≥85%), 3,968 loci (≥25%), 4,241 loci (≥15%), 4,820 loci (≥5%), and 10,704 loci on cluster identification. wgMLST profiling based on various numbers of loci performed equivalently in discerning the outbreak isolates from the non-outbreak isolates for the panel of isolates from the Minnesota Department of Health. However, as the number of loci used in profiling increased, we observed a higher number of loci variations among isolates within an outbreak. Whether wgMLST profiling based on more target genes can provide better resolution for identifying outbreak isolates requires further assessment.

In the present study, the pan-genome allele database was constructed with *S.* Enteritidis genomes, and only the core genes were applied for wgMLST profiling. However, it is important to consider whether this *S.* Enteritidis pan-genome allele database can be applied for wgMLST profiling of other *Salmonella* serovars. One would expect that the core genes of *S.* Enteritidis should also exist in other *Salmonella* serovars; therefore, a pan-genome allele database constructed from genomes of a certain serovar such as *S.* Enteritidis, should also be applicable for wgMLST profiling of other serovars. Our preliminary test found that wgMLST profiling using the *S.* Enteritidis pan-genome allele database could discriminate between three *S.* Typhimurium infection outbreaks. Because the *Salmonella* genus consists of a wide variety of serovars (>2,600 serovars), constructing a universal *Salmonella* pan-genome allele database applicable to all serovars is necessary for public health laboratories.

The *S*. Enteritidis pan-genome allele database was constructed using the PGAdb-builder (Liu et al., 2016). The program grouped proteins that shared ≥95% amino acid sequence identity as a gene cluster (a locus). PGAdb-builder doesn’t provide options for users to adjust the sequence coverage, thus alleles of a locus might have great length differences (i.e., the shorter allele could be only a small portion of the longest one). In this study, the longest allele “nucleotide” sequence of each locus in the database is chosen as reference for wgMLST profiling. In the profiling process, putative allele has to have ≥90% length coverage and ≥90% sequence identity with the reference allele that allows to exclude alleles with variations results from a large deletion or insertion. However, the use of the longest allele of each locus as reference may not be the best choice. Assume the longest allele sequence of a particular locus is 1,000 bp, putative allele sequences less than 900 bp will be excluded. In comparison, when a reference of 800 bp is used, only putative allele sequences less than 720 bp will be excluded. Since *S*. Enteritidis is a very clonal organism, alleles of a gene should have only little variations in length. To choose the longest allele sequence as reference should not be a problem for the clonal *S*. Enteritidis but could exclude most alleles of a gene for a more diversified organism. To choose a sequence with a length that occurs most frequently in the alleles of a gene (i.e., mode) as reference may be more appropriate for a more diversified organism.

## Conclusion

An *S.* Enteritidis pan-genome allele database has been constructed, and the core genes can be used as target genes for wgMLST profiling of isolates. wgMLST profiles are small in size and portable and may be readily standardized and compared across laboratories; therefore, wgMLST may be a superior WGS-based subtyping tool for disease surveillance and outbreak investigation. The database and tools used for wgMLST profiling and cluster analysis of wgMLST profiles have been installed on a website^4^ for public use.

## Author Contributions

Y-YL and C-CC contributed equally to construction of the wgMLST database and the web server and development of tools. C-SC designed the study, analyzed and interpreted the data and drafted the manuscript. All authors approved the final version.

## Conflict of Interest Statement

The authors declare that the research was conducted in the absence of any commercial or financial relationships that could be construed as a potential conflict of interest.

## Footnotes

1 http://www.genomicepidemiology.org/

2 http://www.ncbi.nlm.nih.gov/assembly/

3 http://www.ncbi.nlm.nih.gov/bioproject/PRJNA237212/

4 http://wgmlst.imst.nsysu.edu.tw
