# Supplementary material for: Construction of a Pan-Genome Allele Database of Salmonella enterica Serovar Enteritidis for Molecular Subtyping and Disease Cluster Identification
Source: Front Microbiol. 2016 Dec 15;7:2010. doi: 10.3389/fmicb.2016.02010 (PMC5156723; doi:10.3389/fmicb.2016.02010)
Supplement: Supplementary file 1 [file Table_1.PDF]

Table S1. GenBank Assembly accession numbers for 340 *Salmonella enterica* serovar Enteritidis genomes and GenBank SRA accession numbers for 55 genomes used for construction of a pan-genome allele database.

GCA_000009505.1	GCA_000329605.1	GCA_000336115.1	GCA_000623915.1
GCA_000280075.1	GCA_000329625.1	GCA_000414765.1	GCA_000623935.1
GCA_000280095.1	GCA_000329645.1	GCA_000414785.1	GCA_000623955.1
GCA_000280115.1	GCA_000329665.1	GCA_000414805.1	GCA_000623975.1
GCA_000280135.1	GCA_000329685.1	GCA_000414825.1	GCA_000623995.1
GCA_000280155.1	GCA_000329705.1	GCA_000414845.1	GCA_000624015.1
GCA_000280175.1	GCA_000329725.1	GCA_000414865.1	GCA_000624035.1
GCA_000280195.1	GCA_000329745.1	GCA_000414885.1	GCA_000624055.1
GCA_000280215.1	GCA_000329765.1	GCA_000414905.1	GCA_000624075.1
GCA_000280235.1	GCA_000329785.1	GCA_000414925.1	GCA_000624095.1
GCA_000280255.1	GCA_000329805.1	GCA_000465535.1	GCA_000624115.1
GCA_000280275.1	GCA_000329825.1	GCA_000505105.1	GCA_000624135.1
GCA_000280295.1	GCA_000329845.1	GCA_000612325.1	GCA_000624155.1
GCA_000280315.2	GCA_000329865.1	GCA_000623055.2	GCA_000624175.1
GCA_000280335.1	GCA_000329885.1	GCA_000623075.1	GCA_000624195.1
GCA_000280355.1	GCA_000329905.1	GCA_000623095.1	GCA_000624215.1
GCA_000280375.1	GCA_000329925.1	GCA_000623115.1	GCA_000624235.1
GCA_000280395.1	GCA_000329945.1	GCA_000623135.1	GCA_000624255.1
GCA_000280415.1	GCA_000329965.1	GCA_000623155.1	GCA_000624275.1
GCA_000280435.1	GCA_000329985.1	GCA_000623175.1	GCA_000624295.1
GCA_000280455.1	GCA_000330005.1	GCA_000623215.1	GCA_000624315.1
GCA_000280475.1	GCA_000330025.1	GCA_000623235.1	GCA_001114305.1
GCA_000328985.1	GCA_000330045.1	GCA_000623255.1	GCA_001122105.1
GCA_000329005.1	GCA_000330065.1	GCA_000623275.1	GCA_001130285.1
GCA_000329025.1	GCA_000330085.1	GCA_000623295.1	GCA_001131605.1
GCA_000329045.1	GCA_000330105.1	GCA_000623315.1	GCA_001138325.1
GCA_000329065.1	GCA_000330125.1	GCA_000623335.1	GCA_001145065.1
GCA_000329085.1	GCA_000330145.1	GCA_000623355.1	GCA_001151825.1
GCA_000329105.1	GCA_000330165.1	GCA_000623375.1	GCA_001155045.1
GCA_000329125.1	GCA_000330185.1	GCA_000623395.1	GCA_001163605.1
GCA_000329145.1	GCA_000330205.1	GCA_000626175.1	GCA_001166405.1
GCA_000329165.1	GCA_000330225.1	GCA_000626195.1	GCA_001168305.1
GCA_000329185.1	GCA_000330245.1	GCA_000626215.1	GCA_001185215.1
GCA_000329205.1	GCA_000330265.1	GCA_000626235.1	GCA_001185245.1
GCA_000329225.1	GCA_000330285.1	GCA_000626255.1	GCA_001238365.1
GCA_000329245.1	GCA_000330305.1	GCA_000626275.2	GCA_001240255.1
GCA_000329265.1	GCA_000330325.1	GCA_000626295.1	GCA_001240265.1
GCA_000329285.1	GCA_000330345.1	GCA_000626315.1	GCA_001243345.1
GCA_000329305.1	GCA_000625255.1	GCA_000626335.1	GCA_001245355.1
GCA_000329325.1	GCA_000625275.1	GCA_000626355.1	GCA_001245855.1
GCA_000329345.1	GCA_000625295.1	GCA_000626375.1	SRS569673
GCA_000329365.2	GCA_000625315.1	GCA_000626395.1	SRS569674
GCA_000329385.1	GCA_000625335.1	GCA_000626415.1	SRS569675
GCA_000329405.1	GCA_000625355.1	GCA_000626435.1	SRS569676
GCA_000329425.1	GCA_000625375.1	GCA_000626455.1	SRS569677

GCA_000624335.1	GCA_000625395.1	GCA_000626475.1	SRS569679
GCA_000624355.1	GCA_000625415.1	GCA_000626495.1	SRS569680
GCA_000624375.1	GCA_000625435.1	GCA_000626515.1	SRS569681
GCA_000624395.2	GCA_000625455.1	GCA_000626535.1	SRS569682
GCA_000624415.1	GCA_000625475.1	GCA_000626555.1	SRS569683
GCA_000624435.1	GCA_000625495.1	GCA_000626695.1	SRS569685
GCA_000624455.1	GCA_000625515.1	GCA_000750215.1	SRS569692
GCA_000624475.1	GCA_000625535.1	GCA_000750255.1	SRS569693
GCA_000624495.1	GCA_000625555.1	GCA_000750295.1	SRS569694
GCA_000624515.1	GCA_000625575.1	GCA_000750335.1	SRS569695
GCA_000624535.1	GCA_000625595.1	GCA_000750375.1	SRS569696
GCA_000624555.1	GCA_000625615.1	GCA_000750395.2	SRS569699
GCA_000624575.1	GCA_000625635.1	GCA_000750415.2	SRS569701
GCA_000624595.1	GCA_000625655.1	GCA_000750435.1	SRS569702
GCA_000624615.1	GCA_000625675.1	GCA_000750455.1	SRS569703
GCA_000624635.1	GCA_000625695.1	GCA_000750475.1	SRS569704
GCA_000624655.1	GCA_000625715.1	GCA_000750495.1	SRS569705
GCA_000624675.1	GCA_000625735.1	GCA_000754375.1	SRS569706
GCA_000624695.1	GCA_000625755.1	GCA_000831025.1	SRS569707
GCA_000624715.1	GCA_000625775.1	GCA_000831045.1	SRS569708
GCA_000624735.1	GCA_000625795.1	GCA_000968775.1	SRS569710
GCA_000624755.1	GCA_000625815.1	GCA_000968795.1	SRS569711
GCA_000624775.1	GCA_000625835.1	GCA_000973935.1	SRS569712
GCA_000624795.1	GCA_000625855.1	GCA_000973955.1	SRS569714
GCA_000624815.1	GCA_000625875.1	GCA_000973985.1	SRS569716
GCA_000624835.1	GCA_000625895.1	GCA_000974045.1	SRS569717
GCA_000624855.1	GCA_000625915.1	GCA_001086425.1	SRS569718
GCA_000624875.1	GCA_000625935.1	GCA_001087165.1	SRS569719
GCA_000624895.1	GCA_000625955.1	GCA_001099405.1	SRS569721
GCA_000624915.1	GCA_000625975.1	GCA_001102865.1	SRS569722
GCA_000624935.1	GCA_000625995.1	GCA_000623415.1	SRS569723
GCA_000624955.1	GCA_000626015.1	GCA_000623435.1	SRS569724
GCA_000624975.1	GCA_000626035.1	GCA_000623455.1	SRS569739
GCA_000624995.1	GCA_000626055.1	GCA_000623475.1	SRS569744
GCA_000625015.1	GCA_000626075.1	GCA_000623495.1	SRS569751
GCA_000625035.1	GCA_000626095.1	GCA_000623515.1	SRS569752
GCA_000625055.1	GCA_000626115.1	GCA_000623535.1	SRS569753
GCA_000625075.1	GCA_000626135.1	GCA_000623555.1	SRS569755
GCA_000625095.1	GCA_001103165.1	GCA_000623575.1	SRS569758
GCA_000625115.1	GCA_000330365.1	GCA_000623595.1	SRS569759
GCA_000625135.1	GCA_000330385.1	GCA_000623615.1	SRS569765
GCA_000625155.1	GCA_000330405.1	GCA_000623635.1	SRS569766
GCA_000625175.1	GCA_000330425.1	GCA_000623655.1	SRS569790
GCA_000625195.1	GCA_000330445.1	GCA_000623675.1	SRS569794
GCA_000625215.1	GCA_000330465.1	GCA_000623695.1	SRS569795
GCA_000625235.1	GCA_000330525.1	GCA_000623715.1	SRS569796
GCA_000626155.1	GCA_000330545.1	GCA_000623735.1	SRS569798
GCA_000329445.1	GCA_000330565.1	GCA_000623755.1	SRS569799

GCA_000329465.1	GCA_000330585.1	GCA_000623775.1	SRS569800
GCA_000329485.1	GCA_000335855.1	GCA_000623795.1	SRS570367
GCA_000329505.1	GCA_000335875.2	GCA_000623815.1	
GCA_000329525.1	GCA_000335895.1	GCA_000623835.1	
GCA_000329545.1	GCA_000336055.1	GCA_000623855.1	
GCA_000329565.1	GCA_000336075.1	GCA_000623875.1	
GCA_000329585.1	GCA_000336095.1	GCA_000623895.1	
